# Endocrine Disorder in Patients With Craniopharyngioma

**DOI:** 10.3389/fneur.2021.737743

**Published:** 2021-12-02

**Authors:** Zihao Zhou, Sheng Zhang, Fangqi Hu

**Affiliations:** ^1^Department of Clinical Medicine, Nanjing Medical University, Nanjing, China; ^2^Department of Neurosurgery, Xuzhou Medical University Affiliated Lianyungang Hospital, Xuzhou, China; ^3^Department of Neurosurgery, Nanjing Medical University Affiliated Lianyungang Hospital, Nanjing, China

**Keywords:** craniopharyngioma, endocrine disorders, pituitary hormone deficiency, treatment, risk

## Abstract

Craniopharyngioma is an intracranial congenital epithelial tumor growing along the pathway of the embryonic craniopharyngeal tube. The main clinical symptoms of patients with craniopharyngioma include high intracranial pressure, visual field defect, endocrine dysfunction, and hypothalamic dysfunction. At present, the preferred treatment remains the surgical treatment, but the recovery of endocrine and hypothalamic function following surgery is limited. In addition, endocrine disorders often emerge following surgery, which seriously reduces the quality of life of patients after operation. So far, research on craniopharyngioma focuses on ways to ameliorate endocrine dysfunction. This article reviews the latest research progress on pathogenesis, manifestation, significance, and treatment of endocrine disorders in patients with craniopharyngioma.

## Introduction

Craniopharyngioma is a rare solid or mixed cystic epithelial tumor in the sellar and suprasellar region, accounting for 2–4% of intracranial tumors, which can be found in each age group (1). Patients with craniopharyngioma frequently exhibited hypothalamic–pituitary axis dysfunction, including growth hormone deficiency (GHD), adrenocortical insufficiency, central hypothyroidism, hypogonadism, precocious puberty, hyperprolactinemia, central diabetes insipidus, and hypothalamic obesity (2). In the 15 years ago, pituitary hormone deficiencies have been reported in 54–100% of patients (3, 4). Recent studies have shown that hormone deficiencies occurs in 65–92% of patients, in which childhood-onset is significantly higher than adult-onset (5–8). The current standard treatment is surgery followed by adjuvant radiation therapy (9). However, patients did not return to comparable life-quality scores as a healthy patient collective even after successful treatment (10). Pituitary hormone deficiency is an essential factor affecting long-term quality of life (11) and is associated with poor outcomes (12). Over the last decade, patients with craniopharyngioma have received increased attention concerning specific aspects of their disease, like hormonal deficiencies (10). This article reviews the latest research progress on pathogenesis, manifestation, significance, and treatment of endocrine disorders in patients with craniopharyngioma. In addition, we provide a new perspective and method for diagnosing, treating, and managing hormone deficiency in patients with craniopharyngioma.

## The Pathogenesis of Endocrine Disorders

In the nineteenth century, an increasing number of young patients exhibited unexplained physical and mental symptoms, including loss or delay of sexual maturity, progressive obesity, abnormal somnolence, and dementia-like behavioral changes. These patients were reported to have large solid cystic tumors, characterized by dilatation in the funnel and third ventricle, exceeding the anatomically intact pituitary gland. Cushing chose the term “craniopharyngioma” to refer to these lesions (13). Recent research indicates that tumor mass effect, surgical invasion, radiotherapy, and pituitary fibrosis contribute to the development of endocrine disorders in patients with craniopharyngioma. Consequently, it is beneficial to understand the pathophysiology of endocrine disorders to develop appropriate treatment programs.

### Primary Tumor

Hypothalamic dysfunction caused by the tumor space-occupying effect is a risk factor for developing endocrine disorders (14), and the damage to the hypothalamus–pituitary system caused by the tumor itself is probably permanent (15). The tumor space-occupying effect is closely related to the origin, location, and growth pattern of tumor. Based on tumor origin and the presence of an arachnoid envelope around the pituitary stalk, Pan established a QST typing system for craniopharyngioma (16). Since Q-type tumors originate below the sellar diaphragm, they are classified as epidural tumors; however, as these tumors grow, suprasellar structures are invaded; in extreme cases, the level of the floor of the third ventricle, or even higher, is affected (17). Because tumors vary in their origin, location, and growth pattern, Q-type tumors are theoretically more likely to oppress the neurohypophysis and attack the hypothalamus–pituitary system. Similarly, craniopharyngiomas originating from the subsellar diaphragm have been linked to an increased risk of pituitary dysfunction (16). In addition, the commonest hormonal deficiency in each craniopharyngioma type based on size is different (18): (1) size ≤ 9 mm: 50% in GHD; (2) size 10–19 mm: 73% in secondary hypothyroidism (SHT); (3) size 20–29 mm: 88% in secondary hypogonadism (SHG); and (4) size ≥30 mm: 86% in SHG. It is obvious that larger and more aggressive tumors are more likely to cause pituitary dysfunction (19), especially when it grows more than 20 mm. In recurrent craniopharyngioma, the recurrence site directly affects the growth pattern of recurrent craniopharyngioma and significantly affects hypothalamus–pituitary function (20).

### Surgery

The surgical treatment strategy of craniopharyngioma remains controversial (21). Because of the high recurrence rate of craniopharyngioma, one opinion advocates radical surgery to remove the tumor completely to prevent recurrence and eventually to perform a higher risk operation (22). Another, more conservative approach is that limited and safer excision is supplemented by radiotherapy or radiosurgery for lesions targeting key important structures, especially the hypothalamus (23). Prieto et al. (24) described the status of 500 patients with postoperative craniopharyngioma: the operation-associated mortality rate was 30%, another 15% of patients suffered from severe sequelae, and most of which were correlated with hypothalamic injury. In the perioperative period of craniopharyngioma, the most serious period of endocrine dysfunction is 1–2 weeks after surgery (8), we deduce that postoperative endocrine dysfunction is delayed, which is related to the cycle of pituitary hormone metabolism. It has also been reported that the proportion of postoperative pituitary hormone deficiency, such as diabetes insipidus (DI), hypothyroidism, GHD, adrenocortical dysfunction, and sexual dysfunction, is higher than those before operation (10). DI is the most frequent endocrinopathy following transsphenoidal surgery or transcranial surgery for craniopharyngiomas (25), whereas it is often transient and can be recovered after drug treatment. Common postoperative long-term complications are hypothalamus–pituitary–thyroid (HPT) axis and hypothalamus–pituitary–adrenal (HPA) axis endocrine dysfunction (8). Craniopharyngioma originates from the sellar region and is anatomically connected to the hypothalamus and pituitary. As a result of cystic degeneration and calcification of tumor, adhesion between tumor and the surrounding tissue is quite common (26). The pituitary stalk connects the pituitary and hypothalamus and is mainly composed of nerve fiber bundles, which are easily injured or even ruptured during operation due to overstretching, resulting in impaired hormone secretion. The incidence and severity of injuries are determined by their location and severity (27). Compared with partial resection, total and subtotal resection are more likely to cause damage to hypothalamus, pituitary, and pituitary stalk (28), which is associated with poor prognosis of neuroendocrine in adults (29). Invasive surgery is associated with a high incidence of postoperative hormone defects but has no impact on anterior pituitary function (30). The type of surgery can also affect endocrine outcomes, the ratio of endocrine dysfunction after craniotomy is significantly higher than that after transsphenoidal surgery (31).

### Radiotherapy

Craniopharyngiomas often involve the anterior part of the third ventricle, and its surrounding structures, such as the hypothalamus, are important. When total resection of craniopharyngiomas is difficult, adjuvant radiotherapy after subtotal resection is an effective method to control tumor growth (32). At present, pituitary dysfunction is the most common complication of radiotherapy and chemotherapy. The largest long-term study of children receiving chemotherapy and radiotherapy (748 participants with an average follow-up period of 27.3 years) revealed that cumulative incidence of growth hormone (GH), thyroid-stimulating hormone (TSH), adrenocorticotrophic hormone (ACTH), and gonadotropin (Gn) deficiency at the age of 40 was 72.4, 11.6, 5.2, and 24.4%, respectively (33). Another study reported that a group of 10 patients with craniopharyngioma who received multiple radiotherapies exhibited hormone defects in cortisol and thyroid axes (34). In addition, during the long-term follow-up of more than 100 pituitary tumors or closely related anatomic tumors, it was discovered that most of the GH and Gn deficiency occurred within 5 years after radiotherapy (35). Xu et al. (36) explained the mechanism of radiotherapy-induced endocrine disorders and verified it in mouse experiments: overactivation of p53 signal pathway can induce growth arrest or apoptosis of living cells, whereas Hippo pathway is necessary to induce apoptosis and reduce cell differentiation during the development. Therefore, by activating p53 pathway and inhibiting Hippo pathway, brain radiotherapy can increase apoptosis, decrease cell proliferation, and eventually cause pituitary injury.

### Pituitary Fibrosis

Pituitary fibrosis is also a factor in developing endocrine disorders in patients with craniopharyngioma, especially in GHD. The fact that ameloblastic craniopharyngioma secretes a range of pro-inflammatory cytokines distinguishes it from other sellar tumors (37). Pro-inflammatory cytokines derived from tumor cells infiltrate the brain tissue surrounding ameloblastic craniopharyngioma, potentially producing an inflammatory microenvironment (38). Local inflammatory environmental responses can result in tissue fibrosis, which impairs organ function (39). A significant positive correlation was reported between GHD and pituitary fibrosis, that the cross-talk between craniopharyngioma cells and pericytes in the pituitary plays a critical function in forming GHD, and that interleukin (IL)-1α activates pericytes through IL-1R1-related signaling pathway and then causes pituitary fibrosis, finally leading to decreased GHD levels in craniopharyngioma (40).

## Pituitary Hormone Deficiency

Pituitary hormones include GH, ACTH, Gn, TSH, oxytocin (OT), antidiuretic hormone (ADH), and so on. These hormone deficiencies are critical in the maintenance of normal physiological function. Unfortunately, such hormones are often deficient in patients with craniopharyngioma, impairing their quality of life.

Commonly used methods for determining hormones are radioimmunoassay and chemiluminescent immunoassays. Diagnostic criteria for hypopituitarism: (1) gonadal axis dysfunction: testosterone (TEST) decreased with abnormal follicle-stimulating hormone (FSH), luteinizing hormone (LH), and prolactin (PRL) levels in adult male patients, estradiol (E2) decreased with normal or decreased FSH and LH or abnormal PRL in female patients, and decreased FSH and LH in children (41). (2) The function of GH axis decreased: the insulin growth factor-1 (IGF-1) of the corresponding sex and age group decreased, or when the blood glucose of insulin tolerance test (ITT) was <400 mg/L, the GH was <5 μg/L (42, 43). (3) Central hypothyroidism: the decrease of free thyroxine (FT4) was accompanied by normal or decreased thyrotropin (44). (4) Central adrenocortical dysfunction: when cortisol (COR) <30 μg/L in the morning, or ITT blood glucose <400 mg/L, COR <200 μg/L (45). And (5) DI is defined as the concomitant presence of inappropriate hypotonic polyuria (urine output >3 L/24 h and urine osmolality <300 mOsm/kg) in the presence of high or normal serum sodium (46). When IGF-1 was normal or morning COR > 30 μg/L and <180 μg/L, ITT was performed to determine GH axis function and cortisol reserve function (47, 48).

In the related literature reports, the incidence of various hormone defects in patients with craniopharyngioma is slightly different. We searched PubMed for keywords such as craniopharyngioma, endocrine, and hormone, and the subjects were identified as children or adults, and 11 related literatures (2, 8, 10, 11, 14, 20, 49–53) were obtained. We sorted out the literature to find out the incidence of endocrine dysfunction in craniopharyngioma (Table 1). The most affected hormones in children were GHD (62%) > HPT (60%) > HPA (54%) > DI (42%) > HPG (41%). The most affected hormones in adults were HPT (56%) > HPG (48%) > GHD (38%) > DI (34%) > HPA (33%).

**Table 1:** The reports of endocrine dysfunction in the literature.

**References**	**Object**	**Time**	**Number**	**GHD**	**HPG**	**HPT**	**HPA**	**DI**
Qi et al. (8)	Children	2001–2012	98	75 (77%)	38 (39%)	46 (47%)	53 (54%)	29 (25%)
	Adults	2001–2012	114	39 (34%)	63 (55%)	35 (31%)	37 (33%)	26 (27%)
Qi et al. (49)	Children	1996–2012	109	52 (48%)	–	50 (46%)	35 (32%)	24 (22%)
Tan et al. (50)	Children	1973–2011	185	147 (80%)	118 (64%)	140 (76%)	127 (69%)	115 (62%)
Wijnen et al. (11)	Children	1978–2015	63	31 (53%)	10 (56%)	19 (32%)	17 (28%)	4 (7%)
	Adults	1978–2015	65	17 (27%)	39 (61%)	35 (56%)	25 (39%)	4 (6%)
Bao et al. (20)	Children	1997–2009	20	14/18 (78%)	14 (70%)	8 (40%)	11 (55%)	5 (25%)
	Adults	1997–2009	32	25/27 (93%)	20 (63%)	7 (22%)	17 (53%)	11 (34%)
Mende et al. (10)	Adults	Not mentioned	148	41/135 (30%)	69/136 (51%)	106/136 (78%)	96 (71%)	70/135 (52%)
Guo et al. (14)	Children	2011–2016	185	–	90 (49%)	130 (70%)	115 (62%)	–
Huang et al. (2)	Children	1995–2019	35	–	–	10/26 (39%)	7/17 (41%)	5 (14%)
Sun et al. (51)	Adults	2012–2015	20	–	10 (50%)	12 (60%)	–	12 (60%)
Sowithayasakul et al. (52)	Children	2015–2016	17	8 (47%)	–	11 (65%)	–	–
	Adults	2015–2016	19	16 (84%)	–	14 (74%)	–	–
Boekhoff et al. (53)	Children	2007–2014	215	100 (46.5%)	47 (21.9%)	138 (64.2%)	137 (63.7%)	30 (14.0%)
Total	Children	–	924	427/688 (62%)	317/766 (41%)	552/918 (60%)	486/892 (54%)	212/510 (42%)
	Adults	–	398	138/360 (38%)	176/367 (48%)	209/376 (56%)	175/525 (33%)	123/366 (34%)

*GHD, growth hormone deficiency; HPG, hypothalamus-pituitary-gonad axis; HPT, hypothalamus–pituitary–thyroid axis; HPA, hypothalamus pituitary adrenal axis; DI, diabetes insipidus*.

### Growth Hormone Deficiency

Growth hormone is a peptide hormone secreted by the anterior lobe of human pituitary composed of 191 amino acids and is critical in human physiology, including bone and organ growth, calcium homeostasis, fat decomposition, and weight regulation (54). After our analysis, GHD is more common in children than in adults. Adult GHD is characterized by decreased muscle mass, increased body fat, reduced energy, and well-being (55). GHD in craniopharyngioma children impacts their growth pattern, and their growth rate decreases significantly 1.5–6 years following operation (56). Pituitary dwarfism caused by GHD is the most common pathogenesis in children with short stature, adversely affecting their health (57). GH plays an important role in healthy growth, development, and maintenance of quality of life (58). Once GHD occurs, the GH replacement therapy (GRHT) is the only effective goal-oriented therapy (59). Although there is no clear evidence that GRHT negatively impacts tumor growth, the risk of tumor recurrence remains a critical safety issue (55). Numerous studies have demonstrated that GRHT does not increase the risk of recurrence in patients with craniopharyngioma (55, 60, 61): Smith et al. (60) found that 50 recurrences in these 739 surgically treated patients were recorded, recurrence rate was 6.8%, with a median follow-up time of 4.3 years (range 0.7–6.4 years); Losa et al. (55) researched 89 patients with craniopharyngioma, 49 patients in GRHT, the 5- and 10-year recurrence-free survivals were 92.9 and 84.5%, as compared with 74.5 and 65.2% in the control group, this difference was significant (*p* = 0.024 by the log-rank test), but the significance was only borderline in the sensitivity analysis (*p* = 0.06). A meta-analysis showed that overall craniopharyngioma recurrence rate was lower among children who were treated by GHRT (10.9%, *n* = 3,436) compared with those who were not (35.2%, *n* = 51), the *p*-value comparing the two groups was <0.01 (59).

Clinical trials demonstrate that GRHT positively influences body composition, blood lipids, bone mineral density, and mental health (62). However, even after GRHT, craniopharyngioma mortality remains high; a recent Pfizer International Metabolic Database (KIMS) analysis found that lower IGF-1 measurements at the last terminal sampling were associated with higher mortality, implying that the poor compliance or discontinuation of GRHT in advanced diseases may possess an impact on clinical data in this area (63). In addition, it has been reported that high expression of GH receptor in patients with craniopharyngioma is associated with the short time of postoperative stability. Therefore, if the surgical specimen is craniopharyngioma with high GH expression, GH should be supplemented cautiously (64) even if the tumor volume is uncertain.

### Adrenocorticotrophic Hormone Deficiency

Adrenocorticotrophic hormone is a peptide hormone produced in the pituitary gland that stimulates the formation and secretion of adrenocortical glucocorticoids (especially cortisol) (65). In a non-stressed state, signs and symptoms of adrenal insufficiency may be so subtle that they are not recognized, but they may alternatively include anorexia, nausea, shakiness relieved by eating, hypoglycemia, poor weight gain, poor stamina, or easy fatigability (66). Patients with craniopharyngioma is often identified by symptomatic hyponatremia secondary to ACTH deficiency (67). Adrenocortical dysfunction may greatly impact the rehabilitation of patients, and the hormone replacement therapy is a key step in craniopharyngioma treatment (68). Although the corticosteroid replacement therapy reduces the incidence of hypocortisol (51), risks are concurrently observed. It is stated that in patients with ACTH deficiency, a daily dose of more than 25 mg hydrocortisone is linked to increased mortality compared to lower doses (69). Research by Hammarstrand et al. (70) demonstrated that patients with nonfunctioning pituitary adenoma and adrenal insufficiency receiving a daily dose of more than 20 mg hydrocortisone have increased mortality. The possible explanation is that patients on high doses of (more than 20 mg/day) hydrocortisone replacement have increased total cholesterol, triglycerides, waist circumference, and glycosylated hemoglobin; all these factors are associated with increased cardiovascular morbidity (71). Okinaga et al. (72) reported a high risk of osteoporosis following operation of pituitary tumors, especially craniopharyngiomas. Consequently, it is recommended to reduce the dose of adrenocortical hormone replacement therapy to avoid bone destruction and take measures to protect bone.

### Gonadotropin Deficiency

Following craniopharyngioma growth, the secondary mass effect can oppress the normal pituitary and stalk and lead to a deficiency of important pituitary hormones such as Gn (73), resulting in delayed puberty in children and hypogonadism in adults (4). Clinical evidence of their gonadotropin deficiency includes menstrual disorders, impaired sexual function, and loss of secondary sexual characteristics or lack of evolution in more than half of female patients (15). Gn deficiency may be an early warning sign of cardiovascular risk in adults (74). Pereira emphasized that estrogen deficiency in premenopausal women with craniopharyngioma may increase the risk of cerebrovascular events, requiring appropriate endocrine replacement (75). If left untreated, it will significantly worsen the prognosis (76). Emmert et al. (77) reported a case of secondary Gn dysfunction in patients with craniopharyngioma, suggesting that endocrine pathology may affect gender identity and cause psychological and cognitive impairment in children. It has currently been found that female patients with craniopharyngioma exhibit significantly lower bone mineral density than their matched control group, which may be due to insufficient sex hormone supplementation because sex hormones can mediate the effect on bone and adipose tissue by interacting with neuronal pathways (78). The KIMS database analysis also revealed that sex hormone deficiency might be linked to low standardized bone mineral density (79). In addition, hypopituitarism is associated with pregnancy complications, such as abortion, anemia, pregnancy-induced hypertension, placental abruption, preterm delivery, and postpartum hemorrhage (80). Post-craniopharyngioma pregnancy is rare: 133 female patients with childhood craniopharyngioma were followed up, only six cases became successfully pregnant, but no serious pregnancy complications were observed (81).

### Thyroid-Stimulating Hormone Deficiency

The TSH deficiency can cause central hypothyroidism. Typical hypothyroidism symptoms include cold intolerance, constipation, dry skin, sparse or fragile hair, weight gain, loss of energy, and bradycardia (82). TSH deficiency is critical in diagnosing craniopharyngioma in patients with Hashimoto's thyroiditis (83). The subclinical state of hypothyroidism may have adverse clinical effects on cardiovascular system, lipid, and bone metabolism, and increased mortality, underlining the importance of strict hormone supplementation regulation (84). In patients with TSH and ACTH deficiency, hydrocortisone replacement should precede levothyroxine replacement because levothyroxine increases the metabolic clearance rate of glucocorticoids, and L-thyroxine replacement before hydrocortisone may result in adrenal crisis (82).

### Oxytocin Deficiency

Social and emotional impairment, school dysfunction, and neurobehavioral impairment are highly prevalent in survivors of childhood craniopharyngioma and negatively affect the quality of life. Post-operative deficiency of hormone OT may be the etiology of social/emotional impairment (85). OT is a pituitary neuropeptide hormone synthesized from hypothalamic paraventricular nucleus and supraoptic nucleus (86). OT is critical in regulating a wide range of functions (childbirth and lactation) and complex behaviors (memory, positive social bonds, and stress reduction) (87). Salivary OT levels are lower in patients with anterior pituitary dysfunction than in healthy people (88). The level of OT is positively correlated with psychopathological symptoms, and the level of endogenous OT may be increased in patients with severe depression (89). At present, the effect of OT replacement therapy remains unclear. Gebert et al. (90) believes that the replacement therapy is unnecessary for treating anxiety in patients with craniopharyngioma because patients only exhibit a higher level of state anxiety than the control group (*p* > 0.05). Cook et al. (85) stated that treatment with low-dose intranasal OT resulted in increased desire for socialization and improvement in affection toward family, and the potential of intranasal OT to restore social and behavioral function to pediatric craniopharyngioma survivors should be further explored. Reduced postprandial OT saliva concentrations were observed to be associated with weight problems in childhood-onset craniopharyngioma and adverse eating behavior and symptoms of eating disorders in both childhood-onset craniopharyngioma and controls (91). The OT supplementation may be a therapeutic option for patients with craniopharyngioma with hypothalamic obesity and/or neurobehavioral disorders caused by particular lesions in the anterior hypothalamus (92).

### Antidiuretic Hormone Deficiency

Antidiuretic hormone is a non-peptide hormone produced in the hypothalamus and released into circulation *via* the posterior pituitary in response to an increase in plasma osmotic pressure (93). Central DI occurs when ADH secretion is partially or completely absent (94). DI is a prevalent symptom in patients with craniopharyngioma, with an incidence of 14–18% before operation and 80–93% following tumor resection (95). In DI cases, many electrolytes are lost through the urine, resulting in an out-of-control imbalance of fluid and electrolytes, and in severe cases, they cause slowness, discomfort, and even coma. Severe dehydration and hypernatremia can be fatal (26). Patients with craniopharyngioma with DI have a higher risk of type 2 diabetes, cerebral infarction, severe infection, and a higher mortality rate than the general population (19). The hypernatremia and hyperosmotic state associated with DI have several physiological consequences, including neuronal atrophy, muscle weakness, rhabdomyolysis, decreased ventricular contractility, and impaired glucose utilization, which may be the causes of high mortality in patients with DI (96). Prophylactic administration of ADH can effectively reduce the incidence of early DI and hyponatremia following skull hemangioma microsurgery (97).

## Management

### Perioperative Management

Perioperative management of craniopharyngioma is an important factor affecting neurological function and quality of life of patients (98). A comprehensive preoperative assessment of pituitary hormones should be performed, usually by basic determination (GH/IGF-1, LH, FSH/E2-TEST, TSH/T4, and ACTH/COR) and by routine measurements of 24-h urine volume, 24-h free cortisol, urine-specific gravity, urine osmotic pressure, and electrolytes (99). Certain hormonal changes, such as decreased cortisol, hypothyroidism, and altered water and electrolyte balance, should be corrected before surgery (100).

The onset of polyuria is usually abrupt, occurring within the first 12–24 h after surgery. Acute disorders of water metabolism can manifest in a triphasic pattern (in ~3% of patients): an initial polyuric phase, a subsequent antidiuretic phase (the patients can temporarily concentrate urine and syndrome of inappropriate ADH secretion (SIADH) and hyponatremia develops), and a final polyuric phase that is usually chronic (101). To screen for potential development of postoperative DI and SIADH, patients after surgery undergo assessments of serum sodium and urine-specific gravity every 6 h (102).

Hypernatremia and hyponatremia often occur postoperatively due to DI and SIADH. Treatment strategies for hyponatremia (103): (1) mild or moderate hyponatremia: fluid restriction alone if cause rapidly reversible; otherwise, hypertonic saline solution at 1 ml/kg/h until substantial normalization symptoms. (2) Severe hyponatremia: rapidly increase [Na+] by 4–6 mEq/L with up to three 100-ml boluses of hypertonic saline solution given over 10 min at a time, followed by hypertonic saline solution at 1 ml/kg/h until substantial normalization. If rapid spontaneous correction occurs, it need not be constrained. Treatment strategies for hypernatremia (103): identification of the cause of hypernatremia and its correction. Central DI is usually the cause of hypernatremia, specific replacement therapy for central DI is usually straightforward and primarily aims at ameliorating symptoms (polyuria and polydipsia) by replacing ADH (104). The urine volume is reduced 1–2 h after administration, and the action time varies from 6 to 18 h (105). Nasal feeding purified water is recommended to correct hypernatremia when necessary. The goal of treatment is to adjust the amount of input and the ratio of input fluids to electrolytes to maintain the basic water and electrolyte balance during the acute period after surgery.

The use of glucocorticoids is the most important in the perioperative period of patients with craniopharyngioma. Patients after surgery undergo assessments serum cortisol daily morning (102). At most centers, all patients are given stress doses of hydrocortisone (100 mg IV) or other glucocorticoid at the time of surgery, and this dose is tapered quickly over 2–3 days for a total of about five doses (106–108). Hydrocortisone is then administered orally and tapered down to the preoperative regimen of patients (typically 20 mg in the morning and 10 mg in the evening for hydrocortisone, or 5 mg in the morning and 2.5 mg in the evening for prednisone) (108). Patients who do not have preoperative cortisol deficiency (or are less likely to have one) should generally receive hydrocortisone replacement therapy if their cortisol levels fall below 8 μg/dL twice in a row, with ongoing cortisol replacement needs assessed at follow-up (108). As for the determination of postoperative pituitary hormone, our experience is to perform 3 and 7 days after the operation (increase frequency as necessary). Individualized treatment is taken based on the results of the test. Note that cortisol replacement is superior to thyroid hormone.

### Follow-Up

Follow-up can find tumor recurrence in time, correct and treat water electrolyte, and endocrine state in time. Endocrine, electrolyte, liver and kidney function, and saddle MRI should be performed 14, 30 days, 3, 6 months, and 1 year postoperatively (follow-up frequency should be increased if necessary).

#### Glucocorticoid Supplementation

The glucocorticoid most widely used for the cortisol replacement therapy worldwide is oral hydrocortisone in daily divided doses. At present, the clinical evaluation of the glucocorticoid replacement therapy is mainly based on the clinical evaluation. Too low glucocorticoid doses increase the risk of adrenal crisis and reduce well-being, whereas too high doses increase the risk of complications such as osteoporosis, obesity, and impaired glucose tolerance (109). Given the cortisol production rate and the practicality of use of oral formulations, adults are generally prescribed hydrocortisone 15–20 mg/day (110). Dose adjustment is mainly based on clinical experience and whether patients have new symptoms or remission of symptoms after adjustment (109).

#### Thyroid Hormone Supplement

Compared with primary hypothyroidism, patients with TSH deficiency have more difficulty obtaining the best thyroid hormone replacement because they cannot be guided by serum TSH levels (111). Certain guidelines recommend using free tetraiodothyronine (FT4) as a standard for monitoring central hypothyroidism (112). Daily levothyroxine (L-T4) requirement is 0.8–1.6 mcg/kg, and starting doses typically vary between 50 and 125 mcg/day (113). It is widely recommended that FT4 levels should be maintained within the upper-middle normal range, free triiodothyronine (FT3) should be kept within the normal range, and L-T4 dose should be further adjusted according to clinical reactions and cholesterol levels (111). Combined therapy with L-T4 and liothyronine (L-T3) is not routinely recommended. Evidence from controlled trials has shown no added benefit of combined therapy over L-T4 monotherapy in terms of quality of life, mood, or psychometric measures (114).

#### Growth Hormone Supplement

There is no sign of recurrence 1–2 years after surgery, and GRHT may be considered. As mentioned earlier, GRHT does not promote tumor recurrence. In patients with permanent or confirmed GHD, a starting low rhGH dose (0.01–0.03 mg/day) to be adjusted according to IGF-1 concentrations is also widely accepted (115). Molitch et al. (58) suggest that during GH treatment, patients be monitored at 1- to 2-month intervals during dose titration and semiannually thereafter with a clinical assessment and an evaluation for adverse effects, IGF-1 levels, and other parameters of GH response (including blood pressure, weight and waist circumference, lipid profile, serum glucose, and bone age). The dose should be lowered, or treatment should be discontinued in case of side effects such as arthralgia, headache, and hyperglycemia (115). Some studies have shown that GH replacement caused a lowering of serum free T4 levels and a lowering of serum cortisol levels (116–118); thus, thyroid and adrenal function should be monitored during GH therapy of adults with GHD.

#### Sex Hormone Supplement

Female children are recommended to start the estrogen replacement therapy between the ages of 11 and 12 to mimic average physiology (119). Low doses of estrogen should be used to induce puberty, which is essential to maintain growth potential, and estradiol transdermal is the preferred route (equivalent of 3–7 mcg/day) (120). The estrogen dose is then increased once every 6 months, which will last for about 2–3 years until the girl reaches adult replacement level (121). In male children, pubertal development is induced at age 12, and a slow and gradual increase in serum steroids is obtained. For a monthly intramuscular injection of 25–50 mg of TEST, the dose should be kept as low as possible and increased every 6–12 months until the adult dose is given (122).

Adult male patients with central hypogonadism should be accepted the TEST replacement therapy to reduce fat mass and improve bone mineral density, libido, sexual function, energy levels, sense of well-being, and muscle mass and strength (47). The conventional use of TEST is as follows (122): (1) injectable preparations: TEST esters (enanthate and cypionate) 250 mg can be administered i.m. every 2–3 weeks; (2) oral preparations: multiple daily doses are required (160–240 mg/day in 3–4 doses); and (3) transdermal preparations: the daily doses vary between 5 and 10 g, each delivering 5–10 mg TEST. Hemoglobin, hematocrit, liver function, blood lipids, and prostate antigen should be followed up during treatment.

Hormonal replacement consists of an estrogen component and a progestogen component, in females possessing a uterus (122). Replacement aims to promote and maintain secondary sexual characteristics and to reduce the risk of developing long-term complications such as cardiovascular disease and osteoporosis (123). In premenopausal women, the hormonal replacement therapy as oral estrogen or combined estrogen and progestogen therapy is recommended, assuming that no contraindications are present (47). An average daily dose of 1–2 mg or equivalent of estradiol is required; transdermal preparations are usually applied twice weekly and provided 50–100 μg of E2 daily in a cyclical combination with a progestogen (124). Either sequential transdermal systems with a progestogen component added to estradiol in the second phase of the menstrual cycle (where regular cyclical bleeding is expected) or a continuous combined system with both estradiol and the progestogen delivered throughout is available (125).

#### Diabetes Insipidus

Adequate fluid replacement, treatment of the underlying condition, and desmopressin administration are the mainstays of management (126). We think it is better to control the urine volume at 200 ml/h. The level of blood electrolyte was examined every week within 1 month after operation. The levels of electrolyte and muscle intoxication were checked every month from 1 to 6 months after operation (strengthen the monitoring frequency if necessary). Adjust the appropriate dose and interval according to plasma osmotic pressure and serum sodium concentration. Mild (134–125 mmol/l) hyponatremia can be treated in outpatient setting with fluid restriction and frequent sodium checks, whereas more severe hyponatremia (<125 mmol/l) requires hospitalization with possible short-time use of hypertonic saline or ADH receptor antagonist drugs, being careful to avoid over-correction (127).

## Targeted Therapy

Although the standard treatment for craniopharyngioma, including surgical resection and radiotherapy, can achieve local tumor control, active local treatment often declines quality of life due to permanent neuroendocrine and neuroendocrine defects. The maintenance of quality of life is preferred over complete resection of tumor (128), and reasonable treatment for craniopharyngioma can be supplemented with targeted therapy by reducing the scope of resection or the necessity for follow-up radiotherapy, significantly reducing the incidence of primary diseases and current treatments (129). Targeted genotyping revealed that 95% of papillary craniopharyngiomas had BRAFV600E mutations, and 96% of ameloblastic craniopharyngiomas had CTNNB mutations suggesting that molecular targeted therapy for craniopharyngiomas may be effective (130). Mutations in BRAF kinases activate RAS/RAF/MEK/ERK signaling pathways, which are abnormally activated in many human tumors (131). In addition, *CTNNB1* gene abnormality results in imbalanced Wnt pathway and nuclear β-catenin accumulation, contributing to tumor invasiveness (132). Brastianos et al. (133) reported the treatment of a 39-year-old man having recurrent BRAFV600E craniopharyngioma with dabrafenib (150 mg, twice a day) and trametinib (2 mg, twice a day); after 35 days of treatment, the tumor size was reduced by 85%. Dabrafenib is a BRAF inhibitor and has a good anti-tumor effect in BRAFV600E mutated cancer (131), whereas MEK inhibitor trametinib can enhance the inhibitory effect of BRAF (134). Although Wnt pathway/β-catenin inhibition may be a promising treatment for craniopharyngioma, the potential non-target effect limits its application in current intervention regimens (132). The combined utilization of systemically administered tocilizumab and bevacizumab may be effective in pediatric patients with primarily cystic craniopharyngioma because tocilizumab, a humanized monoclonal antibody, acts against soluble and membrane-bound IL-6R, which has been proved to contain a high level of cystic and solid tumor components in craniopharyngioma (135).

## Summary

Craniopharyngioma is a catastrophic brain tumor, often accompanied by endocrine disorders. Endocrine disorders significantly impair quality of life of patients. Endocrine disorders are caused by primary tumor growth, surgical invasion, radiotherapy, pituitary fibrosis, etc. If left untreated, gonadotropin deficiency in patients with craniopharyngioma increases the risk of poor prognosis; DI increases the risk of mortality and other complications. At present, the replacement therapy remains the treatment option for endocrine disorders, but it must be utilized prudently, and an individualized treatment plan should be developed. The development of targeted therapy may provide a new perspective for improved hormone deficiency.

## Author Contributions

ZZ completed the part of each hormone deficiency. SZ completed the part of mechanisms of hormone deficiency and treatment. FH provided writing ideas and references. All authors contributed to the article and approved the submitted version.

## Funding

Funding from the third level scientific research project of 333 Project of Jiangsu Province Foundation is gratefully acknowledged (BRA2018292).

## Conflict of Interest

The authors declare that the research was conducted in the absence of any commercial or financial relationships that could be construed as a potential conflict of interest.

## Publisher's Note

All claims expressed in this article are solely those of the authors and do not necessarily represent those of their affiliated organizations, or those of the publisher, the editors and the reviewers. Any product that may be evaluated in this article, or claim that may be made by its manufacturer, is not guaranteed or endorsed by the publisher.
